# Comparison of Quercetin and Isoquercitrin’s Anti-Heart Failure Activity via MAPK Inflammatory Pathway and Caspase Apoptosis Pathway

**DOI:** 10.3390/ph18101447

**Published:** 2025-09-26

**Authors:** Ao Guo, Xiangqian Chen, Yuxin Bai, Yulin Dai, Hao Yue

**Affiliations:** 1 Jilin Ginseng Academy, Changchun University of Chinese Medicine, Changchun 130117, China; 2 Integrated Chinese and Western Medicine, Changchun University of Chinese Medicine, Changchun 130117, China; 3 Rehabilitation Medicine Academy, Changchun University of Chinese Medicine, Changchun 130117, China

**Keywords:** isoquercitrin, quercetin, angiotensin II, anti-inflammatory, apoptosis, heart failure

## Abstract

**Background**: Abnormal activation of Angiotensin II (Ang II) serves as a primary trigger for myocardial hypertrophy and cardiac injury. Isoquercitrin (IQ) and Quercetin (Que) possess anti-inflammatory and anti-apoptotic properties, but their protective effects against Ang II-induced cardiac injury remain unclear. This study aimed to investigate the mechanisms and therapeutic efficacy of IQ and Que in heart failure. **Methods**: Cytotoxic effects of IQ and Que on Ang II-induced H9c2 rat cardiomyocyte apoptosis models were assessed in vitro using the CCK-8 assay. Reactive Oxygen Species (ROS) generation and apoptotic fluorescence levels were measured. WB analysis examined protein expression in inflammatory and apoptotic pathways. In vivo heart failure model was established in mice, with cardioprotective effects of IQ and Que evaluated via echocardiography. Molecular docking was employed to analyze ligand–target interactions. **Results**: IQ outperformed Que in promoting cell viability and decreasing ROS. IQ exhibited a more potent inhibitory effect on apoptosis through regulating Bax, Caspase-3, CytoC, and Bcl-2 and demonstrated superior suppression of cardiac inflammation by inhibiting phosphorylation of ERK, JNK, and P38. Compared with Que, IQ more effectively attenuated Ang II-induced cardiac injury by ameliorating reductions in EF% and FS%, suppressing ST-segment elevation, and significantly reducing serum levels of CK-MB, LDH, ANP, BNP, and FFA in a heart failure model. Molecular docking verified stronger binding affinity of IQ for key targets. **Conclusions**: IQ demonstrates superior cardioprotection over Que by regulating MAPK signaling and mitochondrial apoptosis pathways, supporting its potential as a therapeutic candidate for heart failure.

## 1. Introduction

The upregulation of Angiotensin II (Ang II) signaling is closely implicated in the pathogenesis of cardiac hypertrophy and injury [1]. As a potent bioactive molecule and the central effector of the renin–angiotensin system (RAS), elevated Ang II levels exert harmful effects on the heart, including left ventricular (LV) hypertrophy and remodeling [2]. Ang II induces an inflammatory phenotype in cardiac myocytes, leading to cardiac fibrosis and remodeling [3]. In the study of Ang II, it was believed that its myocardial injury mechanism was mainly caused by oxidative stress and induced inflammation, directly damaging the blood vessels and heart [4]. Targeting the Ang II-related inflammatory signaling pathway may provide a novel strategy for preventing hypertension-associated cardiac dysfunction. Flavonoids are secondary metabolites that are widely distributed in various edible plants. Phytochemical studies have found that these compounds and their structural analogs exhibit significant antioxidant and anti-inflammatory properties, making them an important source for new drug development [5]. Isoquercitrin (IQ) is a flavonoid, which is a type of natural product, widely found in fruits and grains [6]. It possesses a range of pharmacological properties, including antioxidant and anti-inflammatory activities [7]. Quercetin (Que) is a natural brass alcohol that is widely distributed in many plants and exists mostly in the form of glycosides [8]. For example, Que can be obtained via hydrolysis [9]. Que has pharmacological effects such as inhibiting inflammation and reducing oxidative stress [10]. Among the flavonoids, Que has attracted much attention because of its significant antioxidant activity and efficient free radical scavenging ability, whereas IQ, which is structurally similar to Que, exhibits higher bioavailability owing to the introduction of glucosides [11]. It has many chemical protective effects against oxidative stress, inflammation, cardiovascular diseases, diabetes, and allergic reactions in vivo and in vitro [12]. The health benefits of flavonoids stem primarily from their antioxidant capacities; specifically, their ability to directly scavenge reactive oxygen species (ROS) serves as a key mechanism that underpins their free radical-neutralizing activity, inhibition of pro-oxidant enzymes, or induction of antioxidant enzymes [13]. Research has found that IQ exhibits significant reactive oxygen species (ROS) scavenging capacity, thereby effectively shielding mesenchymal stem cells (MSCs) from ROS-induced oxidative injury and regulating cell proliferation through cascades like Mitogen-activated protein kinase (MAPK)/NF-κB [14,15]. In addition, Que can easily regulate ROS-induced signaling pathways, including the AMPK and MAPK signaling cascades, to support oxidant defense systems and maintain the oxidative balance [16]. The bioavailability of flavonoid compounds is determined by the composition of aglycones (a structural component of flavonoids) present in plant-based foods [17]. However, Que, a typical flavonoid, has limited solubility, which restricts its broader application. Glycosylation—a strategy widely employed in plant metabolic pathways—effectively enhances the bioavailability of natural products. For instance, IQ has water solubility more than four times greater than that of free Que, leading to not only improved bioavailability but also significantly enhanced biological activity [18]. Therefore, glycosylation of Que with specific sugar moieties represents an effective strategy for increasing its in vivo bioactivity [19].

Research has shown that Mitochondrial dysfunction is also involved in the pathophysiology of heart failure caused by hypertension [20]. Targeted regulation of Ang II-mediated myocardial oxidation and myocarditis may provide novel therapeutic strategies for the treatment of hypertension-induced heart failure [21]. Our previous findings demonstrated that IQ treatment effectively mitigated the 2,2′-azobis[2-methylpropionamidine] dihydrochloride (AAPH)-induced oxidative stress response in 72 hpf larvae, concomitantly decreasing reactive oxygen species (ROS) formation, inhibiting apoptotic activity, and reducing lipid peroxidation, thus constituting a comprehensive protective response [22]. Therefore, this study compared the therapeutic effects and activities of IQ and Que on heart failure from the perspective of anti-inflammatory and antioxidant mechanisms.

## 2. Results

### 2.1. Evaluation of IQ and Que on Ang II-Treated H9c2 Cell Viability, ROS Levels, and Concentration Screening by Cell Counting Kit-8 (CCK-8) and 20,70-Dichlorodihydrofluorescein Diacetate (DCFH-DA) Assays

**Figure 1:**
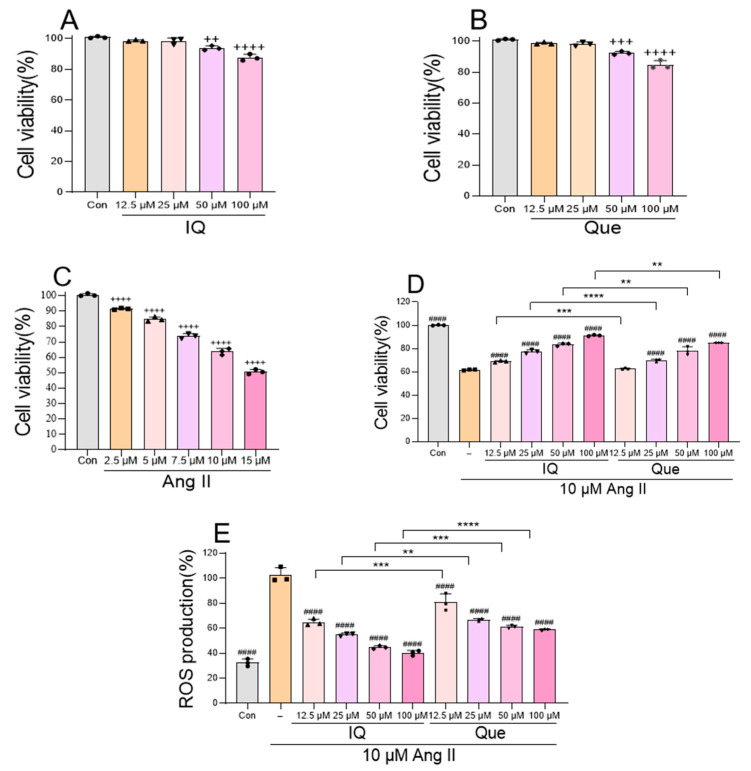
IQ and Que comparably protected H9c2 cells against Ang II induced damage. (**A**) The cytotoxicity of IQ was evaluated in H9c2 cells using CCK-8 assay. (**B**) The cytotoxicity of Que was evaluated in H9c2 cells using CCK-8 assay. (**C**) Ang II concentration optimization in H9c2 cells. (**D**) Protective effects of IQ and Que against Ang II induced cytotoxicity in H9c2 cardiomyocytes. (**E**) IQ and Que attenuate Ang II induced ROS production in H9c2 cells. (n = 3 data were presented as mean ± SEM, ++ *p* < 0.01, +++ *p* < 0.001, ++++ *p* < 0.0001 vs. Con, #### *p* < 0.0001 vs. Ang II, ** *p* < 0.01, *** *p* < 0.001, **** *p* < 0.0001, IQ vs. Que).

Cell viability was assessed using the CCK-8 assay according to the manufacturer’s instructions. CCK-8 assay demonstrated that IQ (100 μM, 24 h) maintained >80% viability in H9c2 cardiomyocytes compared to phosphate-buffered saline (PBS)-treated controls (Con) (Figure 1A), confirming its non-cytotoxicity. Que concentration screening was performed using the same protocol (Figure 1B). Ang II dose-dependently reduced cell viability (Figure 1C), with 10 μM (achieving ~60% viability) selected for subsequent experiments. In Ang II-treated cells (Figure 1D), both IQ and Que mitigated Ang II-induced toxicity in a dose-dependent manner. ROS analysis (Figure 1E) revealed that Ang II significantly increased intracellular ROS levels, whereas IQ and Que treatments attenuated ROS production in a concentration-dependent manner, demonstrating the stronger antioxidant capacity of IQ.

### 2.2. Staining Analysis of IQ and Que on Apoptosis in Ang II Treated H9c2 Cells

**Figure 2:**
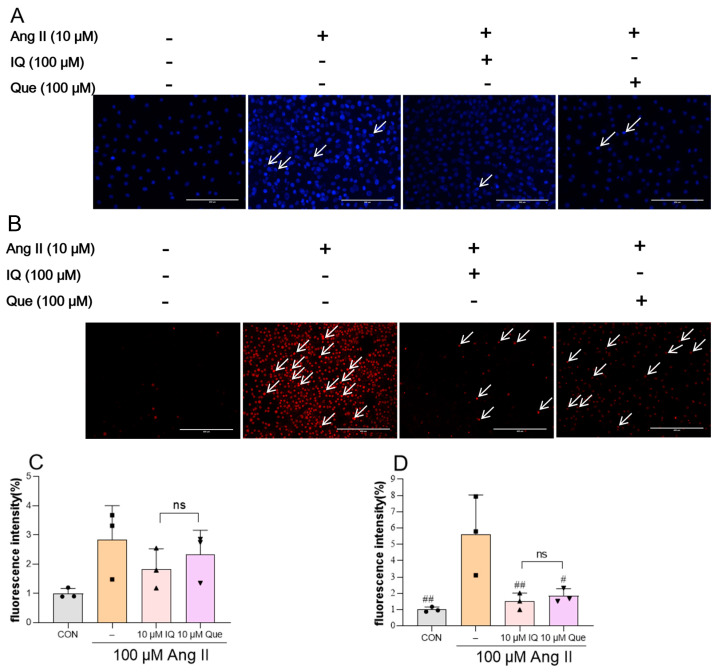
IQ and Que treatments exerted a dose-dependent inhibitory effect on in vitro apoptosis. (**A**) Hoechst 33342 staining of apoptotic cells in IQ and Que treated groups (The scale bar represents 200 μm). (**B**) PI staining for Ang II induced apoptosis in H9c2 cells treated with IQ/Que (The scale bar represents 400 μm). (**C**,**D**) Quantification of Hoechst 33342 and PI fluorescence intensity. Data are presented as mean ± SD. (n = 3 data were presented as mean ± SEM, # *p* < 0.05, ## *p* < 0.01, vs. Ang II, ns, *p* > 0.05, IQ vs. Que).

Nuclear morphological changes and cell viability were assessed using Hoechst 33342 and Propidium iodide (PI) double staining, where Hoechst 33342 stained the nuclei of all cells, and PI selectively labeled the nuclei of dead or late apoptotic cells. Staining with Hoechst 33342 and PI (Figure 2A,B) revealed that the IQ-treated and Que-treated groups exhibited progressively weaker blue granular fluorescence (Hoechst 33342) and red fluorescence (PI) than the Ang II group (Figure 2C,D), the arrow-indicated apoptotic cells demonstrated that the number of dead cells was significantly reduced upon treatment with IQ and Que. Notably, IQ demonstrated stronger anti-apoptotic effects than Que in H9c2 cardiomyocytes.

### 2.3. IQ and Que Alleviate Ang II Induced H9c2 Cell Damage by Regulating Caspase Apoptotic Pathway and MAPK Inflammatory Pathway

**Figure 3:**
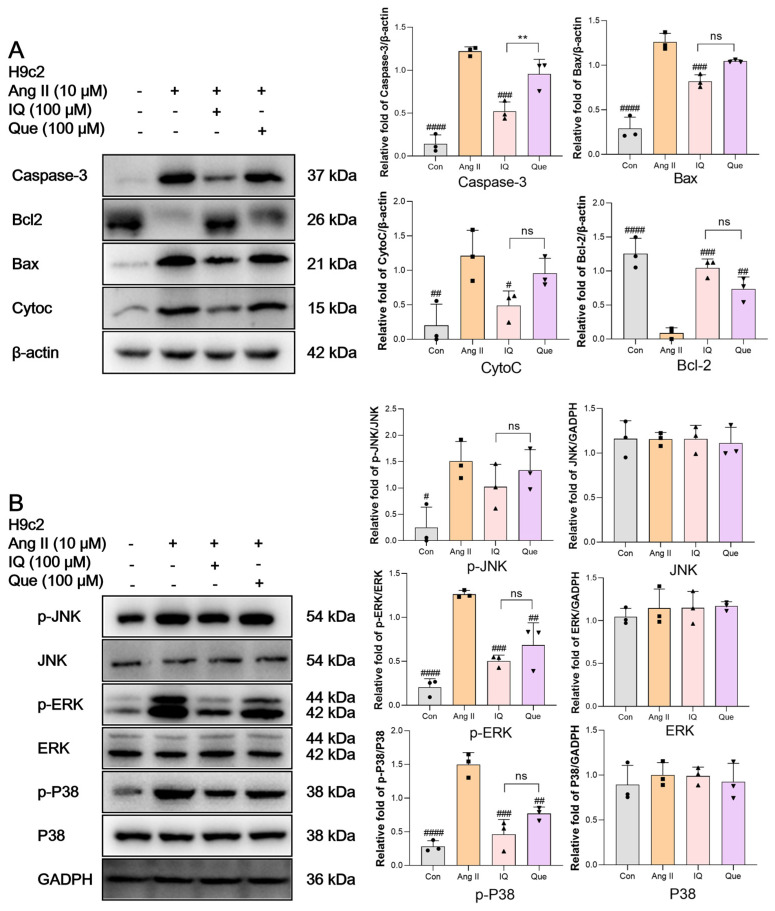
WB analysis of inflammatory and apoptotic proteins in cardiomyocytes treated with IQ and Que in vitro. (**A**) IQ and Que regulate Ang II induced expression of apoptosis related proteins (Caspase-3/Bax/CytoC/Bcl-2) in H9c2 cells. Grayscale quantification of apoptotic markers (Bax/Bcl-2/Caspase-3/CytoC). (**B**) IQ and Que modulate Ang II induced expression of JNK/ERK/P38 and their phosphorylated forms in H9c2 cells. Quantification of inflammatory protein expression by densitometric analysis. Data are presented as mean ± SD. (n = 3 data were presented as mean ± SEM, # *p*< 0.05, ## *p* < 0.01, ### *p* < 0.001, #### *p* < 0.0001, vs. Ang II, ** *p* < 0.01, IQ vs. Que, ns, *p* > 0.05, IQ vs. Que).

Western blot (WB) analysis of apoptotic pathway proteins (Figure 3A) demonstrated that, compared to the Ang II group, both IQ-treated and Que-treated groups showed decreased expression of Cysteine-dependent Aspartate-specific (Caspase-3), Bcl-2-Associated X (Bax), and Cytochrome C (CytoC), along with increased Bcl-2 expression. Notably, IQ exhibited superior anti-apoptotic effects than Que in H9c2 cells. Similarly, the analysis of inflammatory pathway proteins (Figure 3B) revealed that IQ and Que treatment significantly reduced the phosphorylation levels of Extracellular Signal-Regulated Kinase (ERK), c-Jun N-terminal Kinase (JNK), and P38 Mitogen-Activated Protein Kinase (P38) compared to Ang II-treated cells, with IQ demonstrating more potent anti-inflammatory effects than Que.

### 2.4. Quantitative Analysis of Serum Inflammatory Cytokines by Enzyme-Linked Immunosorbent Assay (ELISA) and Body Weight Changes in Mice

**Figure 4:**
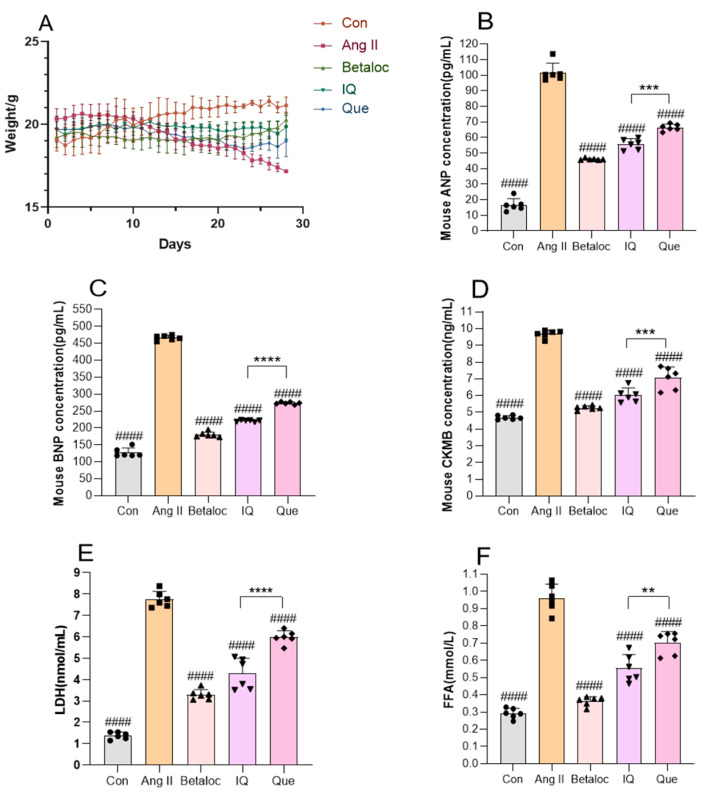
Analysis of body weight changes and serum inflammatory cytokine levels in Ang II-induced mice. (**A**) Body weight changes in mice. (**B**–**F**) Analysis of pro-inflammatory mediators in murine serum samples. (n = 6 data were presented as mean ± SEM, #### *p* < 0.0001 vs. Ang II, ** *p* < 0.01, *** *p* < 0.001, **** *p* < 0.0001 IQ vs. Que).

Chronic Ang II administration induced progressive weight loss, metabolic dysfunction, and elevated cardiac biomarkers Creatine Kinase-MB (CK-MB), Lactate Dehydrogenase (LDH), Atrial Natriuretic Peptide (ANP), Brain Natriuretic Peptide (BNP) and Free fatty acid (FFA) in mice, accompanied by a visible deterioration in coat condition and appetite suppression. Both IQ and Que preventions attenuated these effects in a dose-dependent manner, stabilizing body weight (Figure 4A), and cardiac biomarkers (CK-MB, LDH, ANP, BNP and FFA) normalized to baseline levels (Figure 4B–F), with IQ demonstrating superior anti-inflammatory efficacy compared to Que. Notably, IQ uniquely maintained body weight homeostasis, whereas Que exhibited delayed weight loss.

### 2.5. Echocardiographic and Electrocardiographic Characterization of Ang II Induced Cardiac Dysfunction in Mice

**Figure 5:**
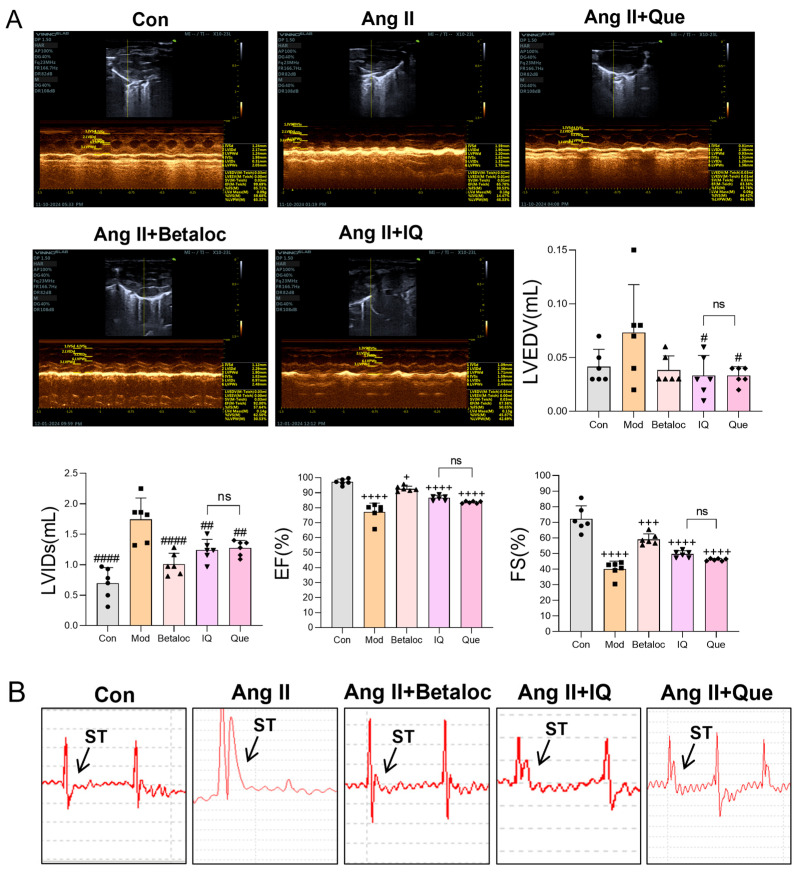
Cardiac function assessment by echocardiography and electrocardiography in Ang II-induced mice. (**A**) Echocardiography in experimental mice. Transthoracic echocardiographic evaluation of cardiac function in mice. (**B**) ECG recordings in mice. (n = 6 data were presented as mean ± SEM, + *p* < 0.05, +++ *p* < 0.001, ++++ *p* < 0.0001 vs. Con, # *p* < 0.05, ## *p* < 0.01, #### *p* < 0.0001 vs. Ang II, ns, *p* > 0.05, IQ vs. Que).

Cardiac ultrasonography (echocardiography) is an important examination method that reflects the cardiac pathophysiological changes [23]. When myocardial injury, infarction, or acute angina occurs, various cardiac parameters become abnormal. In the Ang II group, Left Ventricular Internal Dimension at end-Systole (LVIDs), and Left Ventricular End-Diastolic Volume (LVEDV) were increased, whereas (Ejection Fraction) EF (%) and (Fractional Shortening) FS (%) were decreased (Figure 5A). Based on murine echocardiography, IQ and Que attenuated Ang II-induced increases in LVIDs, and LVEDV, as well as decreases in EF (%) and FS (%). Compared to the Con group, the Ang II group showed significantly lower EF (%) and FS (%). After intervention with IQ and Que, both groups exhibited varying degrees of regulatory effects, with IQ demonstrating superior therapeutic efficacy over Que. ST-segment elevation myocardial infarction (STEMI) is electrocardiographically characterized by ST-segment elevation with upward convexity in two or more contiguous leads [24]. Electrocardiogram (ECG) analysis (Figure 5B) revealed that the Con group exhibited a slowly increasing ST-segment waveform, whereas the Ang II group showed a significant increase in the ST-segment amplitude, confirming successful model establishment. The Betaloc group displayed no significant changes in ST-segment amplitude, whereas the IQ group showed a slight increase, and the Que group exhibited ST-segment elevation.

### 2.6. Cardioprotective Effects of IQ and Que via Apoptosis and Inflammation Pathways

**Figure 6:**
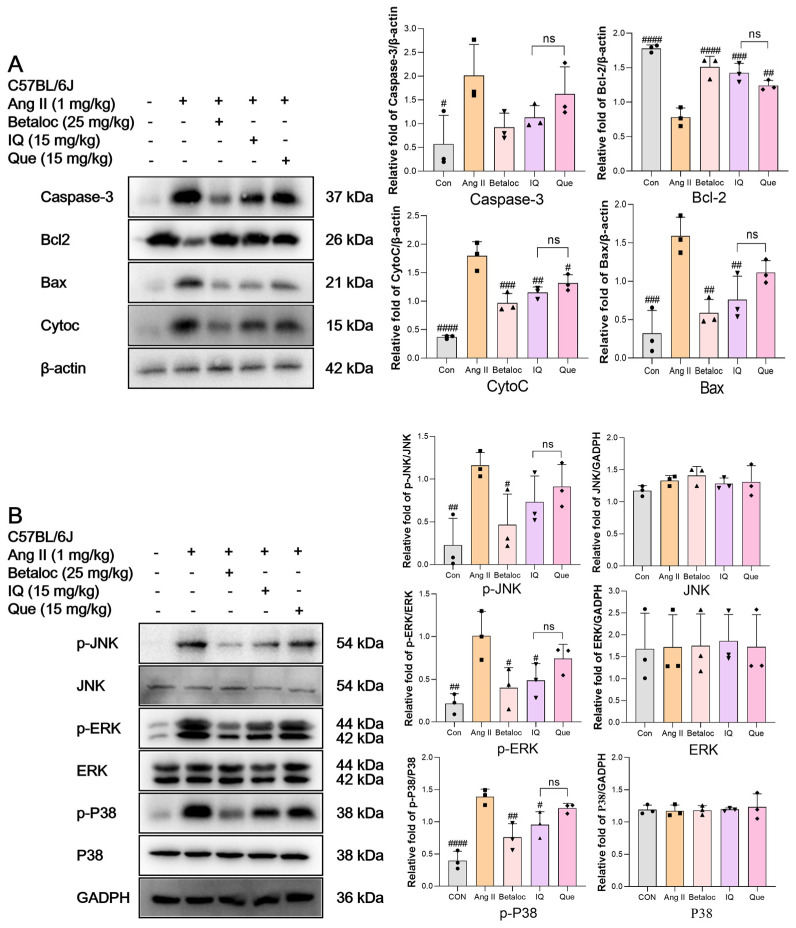
Effects of IQ and Que on apoptotic pathways in Ang II-induced myocardial injured mice. (**A**) IQ and Que regulate Ang II induced apoptosis related proteins (Caspase-3/Bax/CytoC/Bcl-2) in C57BL/6J mice. In vivo quantification of apoptotic protein expression by densitometric analysis. (**B**) Effects of IQ and Que on Ang II-induced JNK/ERK/P38 phosphorylation in C57BL/6J mice. In vivo analysis of inflammatory protein expression. (n = 3 data were presented as mean ± SEM, # *p* < 0.05, ## *p* < 0.01, ### *p* < 0.001, #### *p* < 0.0001, vs. Ang II, ns, *p* > 0.05, IQ vs. Que).

WB analysis revealed that Ang II significantly elevated pro-apoptotic protein expression (Caspase-3, Bax, and CytoC) and reduced anti-apoptotic Bcl-2 levels in the mouse myocardium (Figure 6A). Comparative analysis revealed that Betaloc, IQ, and Que effectively reversed these changes, with Betaloc demonstrating the strongest anti-apoptotic effect, followed by IQ and Que. In the MAPK inflammatory pathway, Ang II markedly increased the phosphorylation of ERK, JNK, and P38 (Figure 6B). All preventions significantly reduced the phosphorylation levels, with IQ showing consistently greater anti-inflammatory efficacy than Que.

### 2.7. Molecular Docking Results of IQ with MAPK Pathway and Caspase Pathway

**Figure 7:**
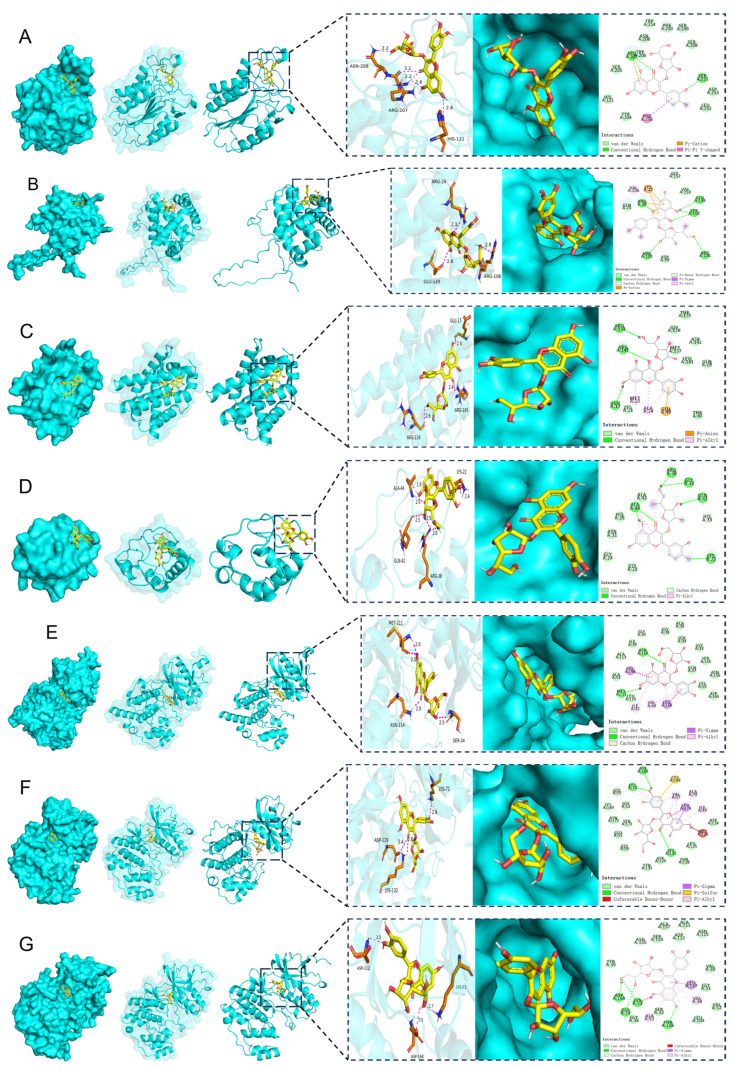
Molecular docking analysis of compounds with core targets. (**A**) IQ with the Caspase-3; (**B**) IQ with the Bcl-2; (**C**) IQ with the Bax; (**D**) IQ with CytoC; (**E**) IQ with JNK; (**F**) IQ with ERK; (**G**) IQ with P38.

Molecular docking validation demonstrated that IQ exhibited favorable binding affinities with MAPK Pathway and Caspase Pathway, with binding energies below −5.0 kcal·mol^−1^, indicating strong interactions. Specifically, IQ showed binding energies of −6.8 kcal·mol^−1^ (Caspase-3) (Figure 7A), −6.2 kcal·mol^−1^ (Bcl-2) (Figure 7B), −6.1 kcal·mol^−1^ (Bax) (Figure 7C), −6.2 kcal·mol^−1^ (CytoC) (Figure 7D), −8.5 kcal·mol^−1^ (JNK) (Figure 7E), −8.1 kcal·mol^−1^ (ERK) (Figure 7F), −8.4 kcal·mol^−1^ (P38) (Figure 7G).

### 2.8. Molecular Docking Results of Que with MAPK Pathway and Caspase Pathway

**Figure 8:**
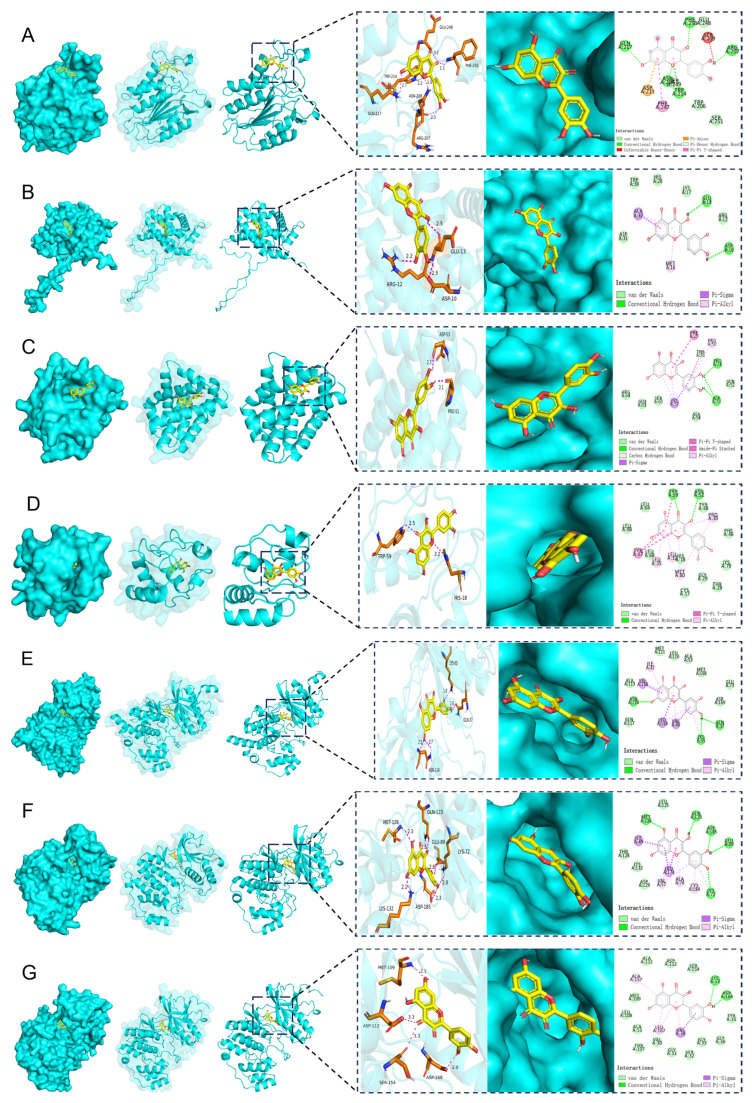
Molecular docking analysis of compounds with core targets. (**A**) Que with the Caspase-3; (**B**) Que with the Bcl-2; (**C**) Que with the Bax; (**D**) Que with CytoC; (**E**) Que with JNK; (**F**) Que with ERK; (**G**) Que with P38.

Molecular docking validation demonstrated that Que exhibited favorable binding affinities with MAPK Pathway and Caspase Pathway, with binding energies below −5.0 kcal·mol^−1^, indicating strong interactions. Specifically, Que showed binding energies of −6.7 kcal·mol^−1^ (Caspase-3) (Figure 8A), −5.5 kcal·mol^−1^ (Bcl-2) (Figure 8B), −5.9 kcal·mol^−1^ (Bax) (Figure 8C), −5.9 kcal·mol^−1^ (CytoC) (Figure 8D), −8.4 kcal·mol^−1^ (JNK) (Figure 8E), −7.9 kcal·mol^−1^ (ERK) (Figure 8F), −7.4 kcal·mol^−1^ (P38) (Figure 8G). These results suggest that IQ forms more stable binding interactions with both target proteins than Que.

## 3. Discussion

Oxidative stress and inflammatory responses are critical factors that contribute to myocardial injury in HF. This study demonstrates that both Que and its glycosylated derivative IQ confer cardioprotection against Ang II-induced damage, primarily through suppressing the MAPK inflammatory pathway and the mitochondrial apoptotic cascade. Notably, our findings reveal that IQ consistently exhibits superior efficacy compared to Que in both in vitro and in vivo models. As natural flavonoid compounds, IQ and Que exhibit multiple beneficial properties, including potent antioxidant and anti-inflammatory effects, which may protect cardiomyocytes from damage. Previous research has shown that both quercitrin (a glycoside of Que) and IQ can eliminate ROS and that IQ has higher ROS-scavenging activity and stronger cell protection than Que. Que and IQ can protect MSCs from damage induced by OH free radicals within the concentration range of 0–100 µg/mL [25]. Apoptosis may be initiated via either the extrinsic death receptor-mediated pathway or the intrinsic mitochondrial pathway, serving as a key mechanism that drives the progression of diverse cardiac diseases to heart failure. The irreversible loss of cardiomyocytes, a central pathological feature of heart failure, directly contributes to the progressive deterioration of cardiac contractile function [26]. The MAPK pathway is a crucial signaling cascade in inflammatory diseases that regulates cytokine expression in response to various pathogens. Activation of Phospho-Extracellular Signal-Regulated (p-ERK), Phospho-p38 Mitogen-Activated Protein Kinase (p-P38), and Phospho-c-Jun N-terminal Kinase (p-JNK) induces inflammatory responses in H9c2 cells [27], MAPK activation promotes cardiomyocyte apoptosis and autophagy, with P38/JNK enhancing apoptosis, while ERK exerts cytoprotective effects. Apoptosis plays a pivotal role in the pathophysiology of heart failure, where MAPK-mediated inflammation and mitochondrial apoptotic pathways (CytoC release and Caspase-3 activation) synergistically drive heart failure progression [28]. Elevated LVIDs reflect diastolic dilation, whereas increased LVESV is correlated with progressive heart failure severity [29].

Previous studies have demonstrated that activation of JNK and p38 upregulates proinflammatory cytokine expression, triggering inflammatory cascades and exacerbating cellular damage. Current findings reveal that IQ exerts anti-apoptotic effects through the suppression of ERK, JNK, and p38 activation, along with their downstream targets Bax, Bcl-2, and Caspase-3 [30]. Experimental evidence reveals that IQ exerts cardioprotection through Bcl-2-mediated preservation of mitochondrial membrane integrity, effectively inhibiting CytoC release, apoptosome formation, and subsequent apoptotic progression [31], while IQ exhibits potent anti-inflammatory effects by suppressing MAPK pathway activation via downregulation of p-P38, p-ERK, p-JNK, and cleaved Caspase-3 expression [20]. Research indicates that enhanced apoptosis results from aggravated oxidative stress and inflammatory responses, while Que alleviates these effects by reducing the levels of pro-apoptotic markers (Caspase-3 and Bax) and significantly increasing the expression of the anti-apoptotic protein Bcl-2 in the hearts of diabetic rats after treatment [32]. The study demonstrates that Que pretreatment significantly suppresses intracellular ROS generation, suggesting its potential inhibition of cardiac fibrosis through ROS pathway modulation. Furthermore, investigation of the effects of Que on cardiac fibroblast proliferation, collagen expression, and MAPK signaling activation revealed that Que inhibited Ang II-induced DNA synthesis, collagen secretion, and phosphorylation of ERK, P38, and JNK. These findings indicated that Que mediates Ang II-induced MAPK pathway activation via ROS suppression. Consequently, Que inactivates MAPK signaling by inhibiting ROS formation, thereby attenuating Ang II-stimulated cardiac fibroblast proliferation and ultimately exerting beneficial effects against myocardial fibrosis [33,34]. Our results align with and yet expand upon existing literature. The critical question of whether the structural difference between Que and IQ translates to a functional advantage in heart failure has remained unanswered. Our research directly addresses this, demonstrating that IQ’s glycosylation confers significant preventive benefits. Beyond confirming a common mechanism, we quantitatively demonstrate IQ’s superior efficacy in preserving cardiac function (EF%, FS%) and maintaining biomarker homeostasis (CK-MB, BNP). Notably, IQ administration effectively prevented Ang II-induced weight loss, revealing its system-wide protective effects and suggesting that its preventive advantage may involve the modulation of additional pathways related to metabolic regulation and inflammatory response.

The enhanced bioactivity of IQ is likely attributable to its improved pharmacological profile. The differences in antioxidant activity between flavonoid glycosides and their aglycones are closely associated with their structural characteristics and metabolic processing [35]. While aglycones exhibit a higher but less stable antioxidant capacity, glycosylation enhances structural stability (particularly of C-glycosides via robust C-C bonds), thereby preserving post-digestive bioactivity despite a reduced initial potency [36], potentially leading to higher bioavailability and more sustained target engagement in vivo. To elucidate the structural mechanisms underlying the superior cardioprotective efficacy of IQ relative to Que, we conducted molecular docking analyses focusing on the interactions of these two compounds with key regulatory proteins in the MAPK and Caspase pathways—pathways that play critical roles in regulating cardiac stress responses and cell survival. Our results revealed that both IQ and Que form stable ligand–protein complexes, as indicated by binding energies of ≤–5.0 kcal·mol^−1^; this threshold is widely recognized to reflect favorable intermolecular interactions, including hydrogen bonding and hydrophobic contacts. Notably, however, IQ exhibited consistently more negative binding energy values across all target proteins compared to Que. This difference holds functional significance, as more negative binding energies typically correlate with higher binding affinity and greater thermodynamic stability of the ligand–target complex. Collectively, these findings establish a structural basis for IQ’s enhanced cardioprotective activity: by forming stronger and more stable interactions with key regulators of the MAPK and Caspase pathways, IQ may more effectively modulate these signaling cascades, ultimately conferring improved protection against cardiac injury. These findings directly link IQ’s structural features to its improved biological activity, underscoring its potential as a preclinical candidate for hypertensive heart failure. This suggests that IQ’s glycosyl group may facilitate more stable and specific interactions with key residues, thereby translating to more potent inhibition of downstream signaling and a stronger overall anti-apoptotic and anti-inflammatory effect, as observed in our functional assays. Notably, these compounds specifically targeted the aspartate residue within the highly conserved DFG motif, which plays a pivotal role in regulating kinase activity. Binding energy calculations demonstrated that IQ exhibited superior inhibitory potential against multiple kinase proteins compared to Que [37]. The positive Con drug Betaloc demonstrated potent efficacy across all parameters, establishing a high benchmark for comparison [38]. Betaloc administration has been demonstrated to reduce the ratio of phosphorylated p38 (p-P38) to total p38 protein and suppress p38 phosphorylation [39]. It also improves stroke volume and cardiac output, which are associated with enhanced BCl-2 function and reduced Caspase-3 expression [40]. Furthermore, treatment with Betaloc inhibits the phosphorylation of p38, thereby attenuating cell death and downregulating Caspase-12 expression in rat cardiomyocytes [41]. The robust cardioprotective effects demonstrated by intraperitoneally administered IQ, which achieved outcomes comparable to Betaloc in reducing inflammatory and apoptotic responses—as indicated by the restoration of key biomarker levels and signaling proteins—support its potential not as a superior agent but as a viable preclinical candidate. Owing to its pleiotropic mechanisms and reproducible efficacy across both in vitro and in vivo models, IQ presents a compelling case for further investigation as a natural-based intervention for hypertensive heart failure. The clinical translation of these findings, however, necessitates a thorough evaluation of its development pathway, particularly a systematic comparison of pharmacokinetic properties, bioavailability, and safety following intraperitoneal versus clinically relevant routes of administration, such as oral delivery. Further studies examining potential synergism between IQ and established heart failure therapeutics may also illuminate its utility as an adjunct or alternative treatment regimen.

Despite our findings, this study has several limitations that should be considered. First, we inferred that the higher efficacy of IQ is due to its better bioavailability; however, we did not directly measure or compare the absorption and metabolism of IQ and Que in our animals. In the absence of data on their actual blood concentrations and cardiac tissue distribution, this remains a plausible hypothesis that needs experimental validation. Second, although we have shown that IQ inhibits the MAPK and apoptosis pathways, the initial molecular targets of IQ—the first proteins it interacts with—are still unknown. Identifying this precise target is a complex but important next step for fully understanding its mechanism. Based on these limitations, we propose the following specific next steps for research. Pharmacokinetic Study: The most immediate experiment is to conduct a straightforward pharmacokinetic study. By measuring the blood levels and heart tissue concentrations of IQ and Que over time after administration in the experimental animals, we can directly test if IQ indeed achieves higher bioavailability and better reaches the heart. Target Identification: Efforts should be made to identify the direct binding target(s) of IQ. Techniques such as cellular thermal shift assays (CETSA) or drug affinity responsive target stability (DARTS) could be employed to find the proteins that IQ directly interacts with, which will help us understand how it starts its protective effects.

## 4. Materials and Methods

### 4.1. Reagent

Bicinchoninic acid (BCA) protein detection kit (item number: PC0020), Hoechst 33342 (item number: C0030), PI (item number: C0080), (Solarbio, Beijing, China), Ang II (item number: S25704), IQ (item number: S33219), Que (item number: S33752), CCK-8 (item number: R22305), DCFH-DA (item number: R22380), RIPA lysis buffer (item number: R32714), (Yuanye, Shanghai, China), sodium dodecyl sulfate polyacrylamide gel electrophoresis preparation kit (SDS-PAGE) (item number: BL522A), electrochemiluminescence (ECL) substrate kit (item number: BL520A), (Biosharp, Beijing, China), p-ERK (item number: 28733-1-AP), ERK (item number: 11257-1-AP), p-JNK (item number: 80024-1-RR), JNK (item number: 51153-1-AP), p-P38 (item number: 28796-1-AP) and P38 (item number: 14064-1-AP), Caspase-3 (item number: 19677-1-AP), Bax (item number: 50599-2-Ig), Bcl-2 (item number: 12789-1-AP), CytoC (item number: 10993-1-AP), GADPH (item number: 10494-1-AP), β-actin (item number: 20536-1-AP), HRP-conjugated Goat Anti-Rabbit IgG(H+L) (item number: SA00001-2), (Proteintech, Chicago, IL, USA), polyvinylidene fluoride (PVDF) membrane (item number: IPVH00010), (Merck, Darmstadt, Germany), fluorescence microscope (Model No. 10119978), (Nikon, Tokyo, Japan), CO_2_ incubator (Device Model: BC-J250), (Thermo, Waltham, MA, USA), electrophoresis apparatus (Serial No. 041BR174116), (BIO-RAD, Hercules, CA, USA). FFA content determination kit (item number: JL-T1313), CK-MB enzyme-linked immunosorbent assay kit (item number: JL12422), BNP enzyme-linked immunosorbent assay kit (item number: JL12884), ANP enzyme-linked immunosorbent assay kit (item number: JL20612) (Jianglai, Shanghai, China), H9c2(2-1) Cell Complete Medium solution (item number: CM-0089), (Pusainuo, Wuhan, China).

### 4.2. Cell Culture

The H9c2 rat cardiomyocyte cell line was purchased from Procell Life Science and Technology Company Limited Wuhan China. H9c2 cardiomyocytes were maintained by adding H9c2(2-1) Cell Complete Medium solution to the cell culture dished in a humidified atmosphere containing 5% CO_2_ at 37 °C. Cells stimulated with 10 μM of Ang II for 24 h.

### 4.3. Cell Viability Assay

The experimental design employed five concentration gradients of IQ and Que treatment groups at 0, 12.5, 25, 50, and 100 μM [22]. The researchers performed the specific operations as follows: First, IQ and Que were dissolved in Dimethyl sulfoxide (DMSO) and then diluted with sterile PBS in a gradient to ensure that the final concentration of DMSO in each treatment group remained below 0.1%. The team seeded H9c2 cells at a density of 1 × 10^4^ cells/well in a 96-well culture plate. Cells were seeded onto culture substrates and maintained in appropriate medium until reaching 80% confluency, with this confluent state typically attained after 20–24 h of growth in a standard cell incubator (37 °C, 5% CO_2_, humidified). Subsequently, IQ and Que solutions at different concentrations were added for intervention treatment. This was followed by the addition of 10 μL of CCK-8 solution to each well, with incubation continued for another 3 h; the total treatment duration included this period without interruption. Absorbance was then measured at 450 nm using a Microplate Reader (Tecan, Männedorf, Switzerland), and the optical density of each well was documented.

### 4.4. ROS Detection Experiment

H9c2 cells were seeded into 96-well plates at a density of 1 × 10^4^ cells per well and cultured under standard conditions (37 °C, 5% CO_2_) until they reached approximately 80% confluence. Subsequently, the cells were treated with IQ or Que at a series of concentrations (0, 12.5, 25, 50, and 100 μM; 10 μL per well) for 1 h. After this pretreatment, cells in the experimental groups were exposed to Ang II (10 μM, 10 μL per well) for 3 h to induce oxidative injury. For the blank control group, an equal volume of PBS was added in place of Ang II to serve as a negative control. The researchers then added 10 μL of DCFH-DA probe solution (diluted 1:1000) to each well. Fluorescence intensity was determined using a microplate reader at an excitation wavelength of 480 nm and an emission wavelength of 530 nm, with measurements conducted under light-shielded incubation conditions.

### 4.5. Hoechst 33342 Staining

In accordance with well-established methodological approaches documented in prior studies [42], H9c2 cells were seeded in a 12-well plate at a density of 5 × 10^4^ cells/well and maintained under standard culture conditions for 24 h prior to drug treatment. The cells were first exposed to gradient concentrations of IQ and Que for 1 h, followed by co-culture with Ang II (10 μM final concentration, 50 μL volume) for an additional 24 h. After treatment, the culture medium was aspirated, and the cells were gently rinsed twice with PBS to remove the detached cells. After 10 µg/mL Hoechst 33342 staining solution was added and the samples were incubated at 37 °C in the dark for 15 min, imaging was conducted at a 200 nm resolution to visualize fine nuclear structures. For this single-staining assay—where Hoechst 33342 fluorescence intensity acts as a quantitative metric—a strictly standardized imaging configuration (encompassing exposure duration, gain, and excitation strength) was preset and kept constant. This ensured that signals from control samples stayed within the linear detection range (without pixel saturation) while maintaining background fluorescence at a stable baseline. All samples, including both control and treatment groups, were processed in a single experimental batch under unchanging imaging and staining conditions (Hoechst 33342 concentration, incubation duration, and temperature), with no adjustments made during acquisition. Images were acquired using a fluorescence microscope (Meyer Instruments, Inc., Houston, TX, USA) fitted with a 40×/1.3 NA oil immersion objective lens, a DAPI filter set (excitation: 387/11 nm, emission: 447/60 nm; Chroma Technology Corp, Windham VT, USA), and a CoolSNAP-Pro color digital camera (Meyer Instruments, Inc., Houston, TX, USA). Image acquisition was conducted at an optical resolution of 200 nm.

### 4.6. Propidium Iodide Staining

H9c2 cells were seeded into 12-well plates at 5 × 10^4^ cells per well and permitted to adhere for 24 h under standard culture conditions. After this attachment phase, cells were pretreated with escalating concentrations of IQ or Que for 1 h. Next, Ang II was added to the culture medium to a final concentration of 10 μM (50 μL volume), and co-treatment proceeded for another 24 h. At the end of treatment, the culture medium was aspirated and discarded, the cells were washed twice with PBS. Researchers then added PI solution (10 µg/mL) and incubated the samples at 37 °C in the dark for 20 min. The PI dye selectively penetrated cells with compromised membranes, producing red fluorescence in dead and late-stage apoptotic cells. Nuclear morphological changes were observed via a red light-excited fluorescence microscope with a 40×/1.3 NA oil immersion objective and CoolSNAP-Pro color digital camera (Meyer Instruments, Inc., Houston, TX, USA), with imaging performed at a resolution of 400 nm. PI staining, all imaging procedures were completed within 30 min to reduce potential artifacts caused by dye toxicity and fluorescence attenuation. For these PI single-staining assays, where fluorescence intensity serves as the quantitative metric, a standardized imaging setup maintaining fixed exposure duration, gain, and excitation strength was adopted to ensure cross-sample data comparability. This configuration kept control sample signals within the linear detection range (without saturation) while preserving consistent background fluorescence levels. All samples were processed in a single experimental batch under uniform imaging and staining conditions, including strictly regulated PI concentration, incubation duration, and temperature. This approach minimized instrument-induced systematic errors and facilitated reliable quantitative comparisons of fluorescence signals across samples, the fluorescence intensity and percentage of positive cells were analyzed using ImageJ (National Institutes of Health, Bethesda, MD, USA; version 1.53t).

### 4.7. Inflammatory Cytokine Levels in Mouse Serum Were Assessed via ELISA

#### 4.7.1. Collection of Blood Samples from C57BL/6J Mice

Mice were anesthetized via intraperitoneal injection of 10% chloral hydrate solution until pain reflexes completely vanished, after which gentle pressure was applied to the neck area to cause eyeball protrusion. The eyeball was then rapidly removed using ophthalmic forceps, allowing blood to drain freely into a pre-chilled centrifuge tube. Following incubation at room temperature until full coagulation occurred, the blood sample was centrifuged at 4 °C for 10–15 min at 2000–3000 rpm. The clear, transparent supernatant was carefully pipetted to serve as the serum sample for subsequent analyses.

#### 4.7.2. Determination of Free Fatty Acid (FFA) Content

For serum sample assays, clarified supernatants were obtained by centrifugation at 1000× *g* for 10 min at 4 °C to remove potential turbidity or particulate matter prior to analysis. The microplate reader was then pre-warmed and equilibrated to 37 °C for 30 min, with the absorbance wavelength set at 546 nm. After all reagents reached room temperature through thermal equilibration, they were carefully aliquoted into the specified wells of the 96-well plate in strict compliance with the experimental protocol.

The assay was conducted following the established enzymatic protocol. Briefly, serum samples, distilled water, and standard solution were dispensed into designated test tubes: 4 µL of sample was added to the assay tube, 4 µL of distilled water to the blank tube, and 4 µL of standard solution to the standard tube. Next, 200 µL of the primary reagent was added to all tubes. After thorough vortex mixing, the reaction mixtures were incubated at 37 °C for 5 min, with initial absorbance (A_1_) measured at 546 nm using a microplate reader. Subsequently, 50 µL of the secondary reagent was added to each tube. The mixtures were vortexed again and incubated at 37 °C for 10 min, after which final absorbance (A_2_) was recorded at the same wavelength. The absorbance change (ΔA) was calculated as ΔA = A_2_ − A_1_ for subsequent quantitative analysis. The concentration of free fatty acids (FFA), expressed in millimoles per liter (mmol/L), was computed using the following Formula:

FFA (μmol) = (C_standard_ × V_2_) × (ΔA_Sample_ − ΔA_Con_) ÷ (ΔA_standard_ − ΔA_Con_) ÷ (V_1_ ÷ V × W) × D = (ΔA_Sample_ − ΔA_Con_) ÷ (ΔA_standard_ − ΔA_Con_) ÷ W × D

where

- C_standard_: The standard has a concentration of 1 mmol/L (equivalent to 1 μmol/mL).
- V_2_: The volume of standard added is 0.004 mL.
- W: Mass (expressed in grams, g).
- D: The dilution factor is 1, indicating no dilution of the samples.
- V_1_: The volume of sample added is 0.004 mL.
- V: The volume of extraction solution used is 1 mL.

#### 4.7.3. Determination of Concentrations of Murine Atrial Natriuretic Peptide (ANP), Brain Natriuretic Peptide (BNP), Lactate Dehydrogenase (LDH), and Creatine Kinase MB Isoenzyme (CK-MB)

In line with the recommended methodology, all reagents and pre-coated strips were equilibrated at ambient temperature for a minimum of 10 min before use. Any unused strips were immediately returned to their original foil pouches with desiccant and stored at 4 °C. Standards and experimental samples—with the latter diluted to reduce matrix interference—were aliquoted in duplicate into designated wells at 100 µL per well. A blank control containing dilution buffer was included in parallel. The sealed plate was then incubated at 37 °C for 1 h. After this incubation, the solution was discarded, and 100 µL of biotin-labeled antibody working solution was added directly to all wells without prior washing. The plate was covered and incubated again at 37 °C for 1 h. Subsequently, the plate was washed three times automatically with 300 µL per well of 1 × washing buffer, with a 1 min incubation between each wash, after which the plate was thoroughly dried on absorbent paper. Next, 100 µL of enzyme-conjugate solution was added to each well, and the plate was incubated at 37 °C for 30 min. Following five additional washes under the same parameters, 90 µL of TMB substrate was added per well, and the plate was incubated at 37 °C for 15 min in the dark. The reaction was stopped by adding 50 µL of stop solution to each well, and the optical density was measured immediately at 450 nm. A standard curve was constructed using serial dilutions of the standard material, and analyte concentrations (ANP, BNP, LDH, and CK-MB) were derived by interpolation, incorporating relevant dilution factors.

### 4.8. Ang II Induced Myocardial Injury Pattern

The Animal Policy and Welfare Committee of the Changchun University of Chinese Medicine approved all animal care and experimental procedures (Approval No. 2024753).

Thirty male C57BL/6J mice (6-week-old, 20–25 g) were acclimatized for 3 days in standard SPF conditions with controlled temperature (21–24 °C), humidity (40–60%), and a 12 h light/dark cycle with ad libitum access to food and water.

Ang II Solution: Fresh Ang II working solution (0.08 mg/mL, ≈80 μg/mL) was prepared by dissolving Ang II powder in sterile physiological saline. Betaloc (the positive Con) was prepared at a concentration of 4 mg/mL in physiological saline immediately before use. Stock solutions (50 mg/mL) were prepared in DMSO and serially diluted with physiological saline to achieve working concentrations (4 mg/mL). The final concentration of DMSO was maintained below 3% (*v*/*v*) to prevent solvent interference. All solutions were sterile-filtered (0.22 μm) and stored at −20 °C protected from light.

After acclimatization, the mice (n = 6/group) received daily intraperitoneal injections for 4 weeks: Con: Normal saline; Ang II, 1 mg/kg; Ang II + Betaloc, 25 mg/kg (administered 45 min post-Ang II); Ang II + IQ, 15 mg/kg (administered 45 min post-Ang II); and Ang II + Que, 15 mg/kg (administered 45 min post-Ang II) [43,44].

### 4.9. Transthoracic Echocardiography

Throughout the experimental period, the body weights of mice were recorded daily. On the evening of day 28, the mice were fasted with water deprived, and echocardiography was conducted on day 29. Myocardial injury or necrosis can result in detectable abnormalities in echocardiographic readings. Before imaging, hair was carefully clipped from the thoracic region using an electric clipper. The mice were then placed on a heated platform to keep their body temperature within the range of 36 °C to 37 °C. Pre-warmed ultrasound gel was evenly applied over the chest, especially the cardiac area, to prevent hypothermia and ensure acoustic coupling—while avoiding air bubbles that might interfere with image quality. Each mouse was stabilized in a supine position with a slight elevation of the head. Using a micromanipulator, the ultrasound probe was precisely aligned perpendicularly to the chest wall and positioned in the parasternal region; direct contact with the sternum was avoided to prevent signal attenuation. A clear parasternal short-axis view was obtained, and the imaging depth and sector width were adjusted based on the actual heart size to ensure full visualization of the left ventricle, including parts of the right ventricular wall [45].

### 4.10. ECG Detection

Mice were anesthetized by intraperitoneal injection of 10% chloral hydrate and positioned in dorsal recumbency on a 37 °C thermostatic pad. Subcutaneous needle electrodes (26 G) were precisely inserted into the right forelimb, left forelimb, and left hindlimb, and secured with medical tape. The electrodes were connected to a bio-signal acquisition system using a three-lead snapshot interface (BL-420N informational biological signal acquisition and processing system). Following a 10 min stabilization period, the investigators acquired continuous ECG recordings. Researchers subsequently selected 12–17 min of artifact-free data segments for quantitative analysis during periods of stable hemodynamics.

### 4.11. Western Blotting (WB)

Protein extraction was conducted independently for H9c2 cells and mouse cardiac tissues. After undergoing the designated treatments, H9c2 cells were washed with ice-cold PBS and lysed in RIPA lysis buffer on ice, with intermittent vortexing for 1 h. The resulting cell lysates were then centrifuged, and the supernatants were harvested. For cardiac tissue samples, homogenization was performed in PBS at a 1:4 (*w*/*v*) ratio. Post-centrifugation, the pellets were resuspended in a modified RIPA lysis buffer (supplemented with phosphatase inhibitors and PMSF) at a 1:10 (*w*/*v*) ratio, followed by lysis on ice and subsequent centrifugation. The protein concentration of all collected supernatants was determined using the BCA assay. These samples were promptly aliquoted and stored at −80 °C to prevent repeated freeze–thaw cycles. All experimental procedures were executed under low-temperature conditions. After electrophoresis initiation, the voltage was adjusted to 120 V once the samples entered the separating gel and was terminated when the tracking dye reached the bottom of the gel with a clear separation of protein markers. Proteins were then transferred to PVDF membranes, blocked with rapid blocking buffer for 5 min, and incubated overnight at 4 °C with primary antibodies (all 1:1000 dilution) against: JNK, ERK, and their phosphorylated forms; P38 MAPK pathway proteins; apoptosis-related proteins (Bax, Bcl-2, Caspase-3, CytoC); and loading controls (β-actin, GAPDH). After washing with Tris buffered saline Tween (TBST), membranes were incubated with horseradish peroxidase-conjugated secondary antibodies for 3 h at room temperature. The working solution of the developing reagent was freshly prepared in accordance with the protocols supplied with the ECL chemiluminescence substrate kit. After the membrane was uniformly incubated with the developing solution, it was promptly placed into a chemiluminescence imaging system. The optimal exposure time was adjusted based on signal intensity to acquire images. Finally, the grayscale values of the target bands were subjected to quantitative analysis using ImageJ software (National Institutes of Health, USA; version 1.53t).

### 4.12. Molecular Docking

The 2D structures of core Protein–Protein Interaction (PPI) network targets were downloaded from the PDB database in pdb format. After removing the water molecules and adding hydrogen atoms, the files were converted to pdbqt format using AutoDockTools (ADT version 1.5.7; The Scripps Research Institute, USA). Molecular docking with potentially active compounds was performed using AutoDock Vina 1.1.2, and PyMOL (Version 2.5.4, Schrödinger, LLC, New York, NY, USA) was used to visualize the strongest binding interactions.

### 4.13. Statistical Analysis

All experimental data were statistically analyzed GraphPad Prism 5.01. In the present study, comparisons among multiple groups were conducted using for one-way analysis of variance (ANOVA) to determine statistical significance. Statistical significance was defined as + *p* < 0.05, ++ *p* < 0.01, +++ *p* < 0.001, ++++ *p* < 0.0001 vs. Con and * *p* < 0.05, ** *p* < 0.01, *** *p* < 0.001, **** *p* < 0.0001, IQ vs. Que and # *p* < 0.05, ## *p* < 0.01, ### *p* < 0.001, #### *p* < 0.0001 vs. Ang II.

## 5. Conclusions

In conclusion, this study demonstrated that Ang II activated the phosphorylation of the MAPK pathway and that IQ showed more pronounced anti-inflammatory effects than Que. Echocardiographic and electrocardiographic analyses revealed that Ang II-treated mice exhibited elevated ST segments along with increased key indicators of impaired left ventricular systolic function. The docking results demonstrated the stable binding of bioactive compounds to key targets, with IQ showing particularly strong interactions with the two proteins. These findings suggest that IQ and Que are promising therapeutic candidates for hypertensive heart failure via modulation of the MAPK pathway.

## Data Availability

All data of relevance are duly incorporated in the article.

## Abbreviations

The following abbreviations are used in this manuscript:

AAPH	2,2′-azobis[2-methylpropionamidine] dihydrochloride
Ang II	Angiotensin II
ANP	Atrial Natriuretic Peptide
Bax	Bcl-2-Associated X
BCA	Bicinchoninic acid
BNP	Brain Natriuretic Peptide
Caspase-3	Cysteine-dependent Aspartate-specific
CCK-8	Cell Counting Kit-8
CK-MB	Creatine Kinase-MB
CytoC	Cytochrome C
DCFH-DA	2,7-Dichlorodihydrofluorescein diacetate
DMSO	Dimethyl sulfoxide
ECG	Electrocardiographic
EF (%)	Ejection Fraction
ELISA	Enzyme-linked immunosorbent assay
ERK	Extracellular Signal-Regulated Kinase
FFA	Free Fatty Acids
FS (%)	Fractional Shortening
GADPH	Glyceraldehyde-3-Phosphate Dehydrogenase
IQ	Isoquercitrin
JNK	c-Jun N-terminal Kinase
LDH	Lactate Dehydrogenase
LVEDV	Left Ventricular End-Diastolic Volume
LVIDs	Left Ventricular Internal Dimension at end-Systole
MAPK	Mitogen-activated protein kinase
P38	P38 Mitogen-Activated Protein Kinase
PBS	Phosphate-buffer saline
p-ERK	Phospho-Extracellular Signal-Regulated
PI	Propidium iodide
p-JNK	Phospho-c-Jun N-terminal Kinase
p-P38	Phospho-p38 Mitogen-Activated Protein Kinase
PPI	Protein–Protein Interaction
Que	Quercetin
ROS	Reactive Oxygen Species
STEMI	ST-segment elevation myocardial infarction
TBST	Tris buffered saline Tween
WB	Western Blot
